# Editorial: Physiological, molecular and genetic perspectives of environmental stress response in plants

**DOI:** 10.3389/fpls.2023.1213762

**Published:** 2023-06-16

**Authors:** Amaranatha R. Vennapusa, Padma Nimmakayala, Mainassara Abdou Zaman-Allah, Pasala Ratnakumar

**Affiliations:** ^1^ Department of Agriculture and Natural Resources, Delaware State University, Dover, DE, United States; ^2^ Department of Biology and Gus R. Douglass Institute, West Virginia State University, Institute, Dunbar, WV, United States; ^3^ International Maize and Wheat Improvement Center (CIMMYT), Harare, Zimbabwe; ^4^ Indian Council of Agricultural Research (ICAR) Indian Institute of Oilseeds Research (IIOR), Hyderabad, India

**Keywords:** environmental factors, abiotic stress, cutting-edge tools, scientific approaches, crop physiological response, genetics

Rapid changes in environmental conditions due to climate change affect agricultural crop production worldwide and create unprecedented challenges. Despite new developments in plant science research and technology, achieving a decent yield under changing environmental conditions continues to be a great challenge (Ehrhardt and Frommer, 2012; Fuglie, 2021). Moreover, modern agriculture has to deal with future climate change and must meet the demands of a rapidly increasing population and food security. With the complexity of different environmental stresses caused by climate change, there is a need for the scientific community to deploy new approaches for understanding the ecological consequences, effect on physiological and agronomic traits, and to uncover the tolerant mechanisms, and identify the candidate targets for crop improvement programs (Yuan et al., 2019; Pascual et al., 2022; Zandalinas and Mittler, 2022; Garg et al., 2023). Understanding the physiological, molecular, and genetic perspectives of environmental stress responses in plants is crucial to cope with the hostile effects of climate change in the present and future scenarios in agriculture. This Research Topic aims to offer the opportunity to plant scientists worldwide to present past and emerging insights related to environmental stresses and crop improvement. Various contributions were received and those published are summarized as follows.

A total of 14 articles were published, of which four dealt with experiments related to moisture stress, three were related to temperature stress and two studies focused on the combined stresses. The remaining three articles were focused on salt stress and two articles were on cadmium stress.

These contributions addressed diverse topics, covering crop responses to experimental drought in combination or not with other stresses (Pinnamaneni et al.; Venkatesh et al.; Chilakala et al.; Tan et al.), drought and waterlogging (Singamsetti et al.), and dehydration stress (Wang et al.). Pinnamaneni et al. focused on the photosynthetic responses of cotton and soybean to varying levels of irrigation regimes (moisture stress) and planting geometries in the USA. The gas exchange and chlorophyll fluorescence parameters provided a better understanding of the photosynthetic component’s regulatory and adaptive mechanisms in response to different moisture levels. This study provided new information about how soybeans preferentially use non-photochemical energy dissipation. In contrast, cotton uses both photochemical and non-photochemical energy dissipation to protect photosystem centers (PSI and PSII) and the electron transport rate under moisture-limited environments. An article on tropical maize hybrids exposed to various moisture regimes (drought, waterlogging, and optimal moisture conditions) with multi-trait-based index selection (MGIDI) by Singamsetti et al. proposed an effective tool for the selection of superior lines based on multiple traits and helps breeders by determining the better strategic choice for climate resilient crop improvement programs. Venkatesh et al. reported a novel approach for multiple gene stacking to improve drought stress tolerance in groundnut. Pyramiding of transgenes belonging to three different transcription factor families, *MuMYB96*, *MuWRKY3*, and *MuNAC4*, from horsegram (*Macrotyloma uniflorum*) was shown to improve water conservation, water mining, and cellular level tolerance traits in groundnut, thus helping to sustain its plant morpho-physiological and biochemical functions under water-limited conditions and providing a viable option to maintain the yield penalty.

Chilakala et al. investigated the influence of drought and high temperature on the incidence and severity of dry root rot disease (caused by *Macrophomina phaseolina*) in chickpeas under extensive on- and off-season field trials and greenhouse conditions. Notably, the water relation studies in this Research Topic are unique, connecting plants’ drought physiology and pathogen-induced physiological state with multiple pieces of evidence from field, greenhouse, and lab studies. The large-scale study by Tan et al. on perennial small tree grapevine species revealed that the combined effect of both heat and drought stresses is more severe compared to the individual stresses. Integrating physiological, transcriptome, and gene network analysis helped to identify candidate genes, complex pathways, and mechanisms involved in combined stresses. This article generated valuable transcriptomic datasets for grapevines and helpful resources for future research and breeding programs.

Persaud et al. evaluated carinata (*Brassica carinata* A. Braun) genotypes under low and high temperatures. The assessed physiological traits and the superior tolerant lines identified in this study are viable resources for plant breeders to develop cultivars that are adaptable to different climatic zones. In rice, Stephen et al. identified potential simple sequence repeat marker (SSR) candidates linked to heat tolerance using bulked segregant analysis (BSA) in the F_2_ population of NERICA-L 44 × Uma. The study by Xu et al. reported the effect of heat stress on the early booting stage and assessed the reproductive, developmental, physiological, and yield parameters. The tolerant lines and the traits identified can be used to understand mechanisms involved in tolerance and for developing heat-tolerant wheat pre-breeding lines.

Ren et al. adopted a multi-omics approach to study molecular responses in sorghum under salt stress. The comparative analysis of transcriptomics, metabolomics, and proteomics aided in identifying the key mechanisms, pathways, genes, proteins, and metabolites responsible for stress tolerance. The novelty of this study is that integrating physiological data with multi-omics analysis provided a precise tool for identifying the molecular mechanisms and key traits to improve salt stress tolerance in sorghum. The study by Kumar et al. focused on deciphering trait-associated physiological alterations under salt stress conditions using pearl millet hybrids and inbred lines. The meticulous stress imposition methods and trait modeling approach helped to identify the crucial morpho-physiological characteristics governing the tolerance mechanisms and grain yield traits under irrigation-induced salinity stress. The identified pearl millet lines can be recommended for enhancing crop resilience to achieve stable production in saline agroecosystems. Ran et al. assessed the salt tolerance of *Salix matsudana* Koidz, which is a climate-resilient tree species. The study reveals the relationship between ion (Na, K, Ca) absorption and photosynthetic response under salt stress.

Yuan et al. reported the global metabolomic profiling of the edible and medicinal plant *Salvia miltiorrhiza* under cadmium (Cd) stress. The identified target metabolites in this study will be a potential source for improving heavy metal stress tolerance in medicinally important crop plants. The review article by Al-Khayri et al. discusses the Cd source, mobility, and toxicity in medicinal plant species and their effect on germination, plant growth, physiological and biochemical characteristics, and molecular aspects, with a focus on the biosynthesis of important secondary metabolites. Further, this review discusses multiple omics and biotechnological approaches, such as genetic engineering and genome editing to ameliorate Cd stress.

The broad range of articles published under this Research Topic offer the opportunity to extend our understanding of the physiological, molecular, and genetic responses of plants to multiple environmental stresses and their interactions. The reported recent advances in scientific approaches, research ideas, and cutting-edge tools will help to better understand how multiple environmental stresses affect crops and identify the best avenues for developing crops with improved climate resilience.

## Author contributions

PR organized the Research Topic together with AV, PN, and MZ-A. AV and PN wrote the first draft of the editorial, and all coauthors edited the final version.
